# Gestational diabetes mellitus and retinal microvasculature

**DOI:** 10.1186/s12886-016-0398-7

**Published:** 2017-01-18

**Authors:** Ling-Jun Li, Michael Kramer, Robyn J. Tapp, Ryan E. K. Man, Ngee Lek, Shirong Cai, Fabian Yap, Peter Gluckman, Kok Hian Tan, Yap Seng Chong, Jia Yu Koh, Seang Mei Saw, Yin Bun Cheung, Tien Yin Wong

**Affiliations:** 1 Singapore Eye Research Institute, Singapore National Eye Centre, 11 Third Hospital Ave, Singapore, 168751 Singapore; 2 Centre for Quantitative Medicine, Duke-NUS Graduate Medical School, 8 College Road, Singapore, 169857 Singapore; 3 Department of Obstetrics and Gynaecology, Yong Loo Lin School of Medicine, National University of Singapore, Singapore, Singapore; 4 Departments of Pediatrics and of Epidemiology, Biostatistics and Occupational Health, McGill University Faculty of Medicine, Montreal, Canada; 5 KK Women’s and Children’s Hospital, Singapore, Singapore; 6 Singapore Institute for Clinical Sciences, Agency for Science, Technology and Research, Singapore, Singapore; 7 Singapore Institute for Clinical Sciences, Growth, Development & Metabolism, Singapore, Singapore; 8 Saw Swee Hock School of Public Health, National University of Singapore, Singapore, Singapore; 9 Department for International Health, University of Tampere, Tampere, Finland

**Keywords:** Retinal microvasculature, Gestational diabetes mellitus, Pregnancy outcomes, Retinal imaging, Retinal microvascular measures

## Abstract

**Background:**

Small-vessel dysfunction may be an important consequence of chronic hyperglycemia. We examined the association between gestational diabetes mellitus (GDM), a state of transient hyperglycemia during pregnancy, and retinal microvascular changes in pregnant women at 26–28 weeks of pregnancy.

**Methods:**

A total of 1136 pregnant women with singleton pregnancies were recruited during their first trimester at two major Singapore maternity hospitals in an on-going birth cohort study. Participants underwent an oral glucose tolerance test and retinal imaging at 26–28 weeks gestation (*n* = 542). We used the 1999 World Health Organization (WHO) criteria to define GDM: ≥7.0 mmol/L for fasting glucose and/or ≥7.8 mmol/L for 2-h post-glucose. Retinal microvasculature was measured using computer software (Singapore I Vessel Analyzer, SIVA version 3.0, Singapore Eye Research Institute, Singapore) from the retinal photographs.

**Results:**

In a multiple linear regression model adjusting for age, ethnicity and maternal education, mothers with GDM had narrower arteriolar caliber (−1.6 μm; 95% Confidence Interval [CI]: −3.1 μm, −0.2 μm), reduced arteriolar fractal dimension (−0.01 Df; 95% CI: −0.02 Df, −0.001 Df;), and larger arteriolar branching angle (1.8°; 95% CI: 0.3°, 3.3°) than mothers without GDM. After further adjusting for traditional risks of GDM, arteriolar branching angle remained significantly larger in mothers with GDM than those without GDM (2.0°; 95% CI: 0.5°, 3.6°).

**Conclusions:**

GDM was associated with a series of retinal arteriolar abnormalities, including narrower caliber, reduced fractal dimension and larger branching angle, suggesting that transient hyperglycemia during pregnancy may cause small-vessel dysfunction.

## Background

Gestational diabetes mellitus (GDM) is defined as glucose intolerance during pregnancy among women without pre-pregnancy diabetes mellitus and is mainly diagnosed in the second or third trimester [1]. Women with GDM are not only at risk of short-term pregnancy complications such as pre-eclampsia, but also have increased long-term risk of obesity, dyslipdemia and type 2 diabetes mellitus (T2DM) [1, 2]. The latter conditions, in turn, are major risk factors for cardiovascular disease [3, 4], perhaps through endothelial and small-vessel dysfunction [5, 6].

Retinal vascular imaging is now a non-invasive tool for assessing the microvascular dysfunction in health and disease [7–9]. T2DM has been linked to a series of abnormalities in retinal vascular measures such as retinal arteriolar narrowing, retinal venular widening, greater retinal vascular tortuosity, and retinal vascular fractal dimension reduction [8]. Such evidence suggests that small-vessel disease might be an important consequence of insulin resistance and chronic hyperglycemia. Whether small-vessel dysfunction is also present in GDM has not been well studied, however, owing to the difficulty in assessing the microvasculature *in vivo*. There has been a lack of work studying changes in the maternal retinal microvasculature during pregnancy, specifically with GDM.

Our group has recently investigated retinal microvasculature changes during pregnancy, with findings suggesting abnormal retinal vascular morphological changes associated with elevated blood pressure, maternal obesity, antenatal depression, and poor sleep quality [10–12]. In this study, we studied the relationship between GDM and retinal vascular changes in expectant mothers, a proxy for small-vessel dysfunction, at 26–28 weeks of pregnancy.

## Methods

### Study population

A total of 1136 women with singleton pregnancies were recruited during their first trimester in a pregnancy/birth ongoing cohort study (Growing Up in Singapore Towards Healthy Outcomes [GUSTO]) from June 2009 to September 2010. The study methodology has been detailed elsewhere [13]. Inclusion criteria for pregnant women were: (1) Singaporean residents aged 18 years and above; (2) attending either KK Women’s and Children’s Hospital (KKH) or National University Hospital (NUH); and (3) intending to deliver and reside in Singapore for the next 5 years. Due to logistical issues, only participants attending KKH (847 out of 1136, 74.6%) were able to undergo retinal examination. Excluding patients not showing up for 26–28 weeks gestation visit or not willing to take retinal examination, a further subset of expectant women (542 out of 847, 64.0%) without personal diabetes history and with both Oral Glucose Tolerance Test (OGTT) and retinal photography results were included in our analyses.

This study was conducted according to the tenets of the Declaration of Helsinki and approved by both SingHealth Centralized Institutional Review Board and the National Health Group’s Domain Specific Review Board. Written informed consent was obtained from all participants during recruitment.

### OGTT and GDM Definitions at 26–28 weeks gestational visit

Testing for GDM was performed using a 75 g oral glucose tolerance test after overnight fasting (8 to 10 h) at 26–28 weeks gestation. We used the 1999 World Health Organization (WHO) criteria to define GDM: ≥7.0 mmol/L for fasting glucose and/or ≥7.8 mmol/L for 2-h post-glucose [14, 15]. Women with GDM were subsequently managed according to standard protocols practiced at both KKH and NUH [14].

### Retinal photography and vessel assessment at 26–28 weeks gestational visit

**Fig. 1:**
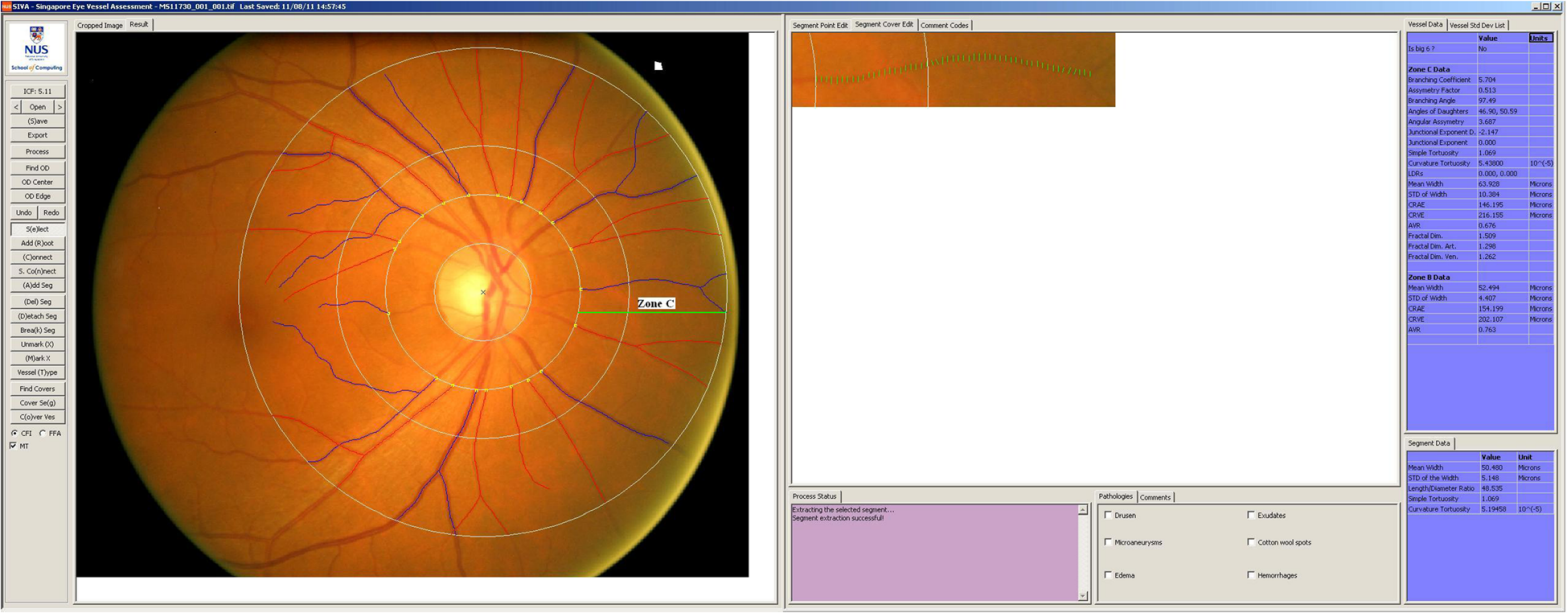
Image of SIVA grading platform. Retinal microvasculature assessment on the grading platform. A screenshot of a computer-assisted program for measurement of new geometrical retinal vascular parameters from retinal fundus photograph. Zone C is marked in SIVA software by 0.5 to 2.0 optic disc diameter away from the margin of optic disc, respectively. All retinal arterioles and venules larger than 25 µm are marked and assessed within zone C

Right eye digital retinal photographs were taken from participants without pharmacological pupil dilation using a 45° non-mydriatic retinal camera (Canon CR-1, 40D SLR digital retinal camera backing; Canon Inc., Japan). The best-quality retinal image centered on the optic disc of each participant was assessed by one trained grader using a semi-automated computer-based program (Singapore I Vessel Assessment [SIVA], version 3.0, Singapore Eye Research Institute, Singapore). This trained grader was blinded to either OGTT results or patient data. Retinal vascular measures were assessed quantitatively at 0.5–2.0 disc diameters (zone C) from the optic disc margin (Fig. 1).

They included the following:

- Retinal vascular caliber was defined as the width of either retinal arterioles or venules. Morphological changes in such caliber (i.e., retinal arteriolar narrowing and/or retinal venular widening) have been linked to systemic conditions such as hypertension and diabetes [7].
- Retinal vascular branching angle was defined as the first angle subtended between two daughter vessels at each bifurcation. Larger vascular branching angle may indicate pathological changes in retinal vascular geometry [7].
- Retinal vascular fractal dimension quantifies the complexity of the branching pattern of the retinal vascular tree. A lower value for the fractal dimension reflects a sparser vascular network and has been observed in diseases such as stroke [16] and hypertension [7].

Intra-grader reliability was assessed in 10% (*n* = 54) of randomly selected retinal photographs from our study, and the intra-class correlation coefficient was above 0.80 for all retinal vascular measures as previously reported [10–12].

### Anthropometric measurements at 26–28 weeks gestational visit

Standing height was measured using the SECA model 213 (Seca, Hamburg, Germany) and weight was assessed using the SECA model 803 (Seca, Hamburg, Germany) according to standardized protocols [12]. Women were asked to remove their shoes and any objects in their pockets, after which both height and weight were measured twice. If the first two measurements differed by ≥1.0 cm for height or ≥200 g for weight, a third measurement was taken to calculate the average. Body mass index (BMI) was defined as weight in kg/(height in m)^2^.

### Questionnaire and clinic interview at 26–28 weeks gestational visit

Questionnaires were administered by trained staff either in English, Chinese, Malay, or Tamil. Information on maternal education and household income, personal history of hypertension, family history of diabetes, past pregnancy history (parity and past GDM), and pre-pregnancy weight was collected. Weight gain at 26–28 weeks gestation was defined as the measured weight at 26–28 weeks gestation minus the recalled weight before pregnancy.

### Statistical analysis

Comparisons of characteristics between GDM and non-GDM participants were analyzed either by Student’s t-test or χ^2^-test. Based on the normal distribution of retinal vascular measures and fasting and 2-h glucose levels, these variables were analyzed as continuous variables. GDM status was analyzed as a binary variable (present/absent).

Multiple linear regression models were constructed to assess the associations between GDM (exposure) and the maternal retinal vascular outcomes mentioned above. Two models were applied in all analyses. Model 1 estimated associations after adjusted for socio-demographic factors, including age, ethnicity, and maternal highest education; Model 2 additionally adjusted for GDM risk factors, including personal history of hypertension, weight gain at 26–28 weeks gestation, family history of diabetes, maternal pre-pregnancy overweight/obese status, history of past GDM, and parity. All statistical analyses were performed using PASW 19.0 (SPSS Inc, Chicago, U.S.). *P* values and 95% confidence intervals (CIs) were presented accordingly. Significant interactions of GDM*age, GDM*ethnicity and GDM*pre-pregnancy overweight/obese status were defined as *P* < 0.1.

## Results

Among the 1136 GUSTO women with an OGTT performed, 542 had further retinal images taken. No statistically significant differences were observed between mothers with and without retinal photography done, except for age (30.4 years vs. 31.2 years, *p* = 0.02) (Table 1). None of the 542 participants had a history of pre-pregnancy diabetes. Of these 542, 88 (16.8%) had developed GDM by 26–28 weeks pregnancy.

**Table 1:** Comparison of baseline characteristics between GUSTO mothers included vs. excluded in this study

Characteristics	Participants		*p* value^*^
	Included (*n* = 542) (mean, SD)	Excluded (*n* = 594) (mean, SD)	
Age (years)	30.5, 5.5	31.2, 4.8	**0.02**
Ethnicity, Chinese	47.1%	52.5%	0.64
Maternal Education, University	25.2%	31.0%	0.91
Household income, >SGD 6000/month	27.6%	26.3%	0.76
History of hypertension, Yes	2.4%	1.8%	0.85
Family history of diabetes, Yes	27.1%	22.8%	0.75
Cigarette smoking history, Yes (%)	10.5%	10.3%	0.40
Alcohol drinking history, Yes	33.8%	32.00%	0.91
Pre-pregnancy weight (kg)	56.8, 11.9	56.9, 11.1	0.98
BMI at 26–28 weeks’ pregnancy (kg/m^2^)	26. 2, 4.6	26.0, 4.7	0.48
gestational diabetes (GDM) onset, Yes	16.2%	17.7%	0.93
CRAE (μm)	121.0, 9.0	120.6, 8.9	0.62
CRVE (μm)	171.4, 12.7	170.0, 12.9	0.18

*Abbreviation*: *GUSTO* Growing Up in Singapore Towards Health Outcomes, *BMI* body mass index, *CRAE* central retinal arteriolar equivalent, *CRVE* central retinal venular equivalent

Bold data mean difference and 95% CI, meaning the *p* value for such estimates are significant

^*^Statistical analysis was performed either by student’s t-test or χ^2^ test

Mothers who developed GDM were significantly older (mean 32.8 vs 30.0 years, *P* < 0.001), had a higher pre-pregnancy BMI (24.4 vs. 22.5 kg/m^2^, *P* < 0.01), experienced lower weight gain by 26–28 weeks gestation (7.6 vs. 8.8 kg, *P* = 0.02), and were more likely to have a family history of diabetes (28.4 vs. 19.4%, *P* = 0.06), and a past history of GDM (8.0 vs. 0.9%, *P* < 0.001) (Table 2). Furthermore, GDM mothers had significantly narrower retinal arteriolar caliber (118.8 vs. 121.3 μm), narrower retinal venular caliber (168.4 vs. 171.9 μm), reduced retinal arteriolar fractal dimension (1.24 vs. 1.26 Df), decreased retinal venular fractal dimension (1.22 vs. 1.23 Df), and larger retinal arteriolar branching angle (84.5 vs. 82.2°) than non-GDM mothers (Table 2).

**Table 2:** Comparison of GUSTO mothers with and without gestational diabetes mellitus (GDM)

Characteristics	GDM (*n* = 88) Mean, SD	Non-GDM (*n* = 454) Mean, SD	*P* value^*^
Maternal characteristics			
Age, years	32.8, 4.9	30.0, 5.4	**<0.001**
Chinese, *n* (%)	49 (55.7%)	232 (51.1%)	**0.03**
Personal history of hypertension, *n* (%)	3 (3.4%)	6 (1.3%)	0.16
Family history of diabetes, *n* (%)	25 (28.4%)	88 (19.4%)	0.06
Past/Current smoking history, *n* (%)	9 (10.2%)	70 (15.5%)	0.20
Past GDM history, *n* (%)	7 (8.0%)	4 (0.9%)	**<0.001**
Maternal education, university, *n* (%)	27 (30.7%)	116 (25.6%)	0.32
Household income > SGD 6000/month, *n* (%)	20 (24.1%)	81 (18.9%)	0.14
Pre-pregnancy BMI (kg/m^2)^	24.4, 4.9	22.5, 3.4	**<0.01**
Weight gain at 26–28 weeks (kg)	7.6, 4.6	8.84, 4.75	**0.02**
Primiparous, *n* (%)	29 (33.0%)	200 (44.1%)	0.14
Baby gender, male (%)	48 (51.5%)	234 (54.6%)	0.80
Maternal retinal vascular measures			
Retinal arteriolar caliber (μm)	118.8, 9,8	121.3, 8.8	**0.02**
Retinal venular caliber (μm)	168.4, 12.8	171.9, 12.6	**0.02**
Retinal arteriolar fractal dimension (Df)	1.24, 0.05	1.26, 0.05	**<0.01**
Retinal venular fractal dimension (Df)	1.22, 0.05	1.23, 0.04	**<0.01**
Retinal arteriolar branching angle (degrees)	84.5, 8.7	82.2, 9.2	**0.03**
Retinal venular branching angle (degrees)	79.5, 10.1	80.5, 9.0	0.34

Bold data mean difference and 95% CI, meaning the *p* value for such estimates are significant

^*^Statistical analysis was performed either by student’s t-test or χ^2^ test

**Fig. 2:**
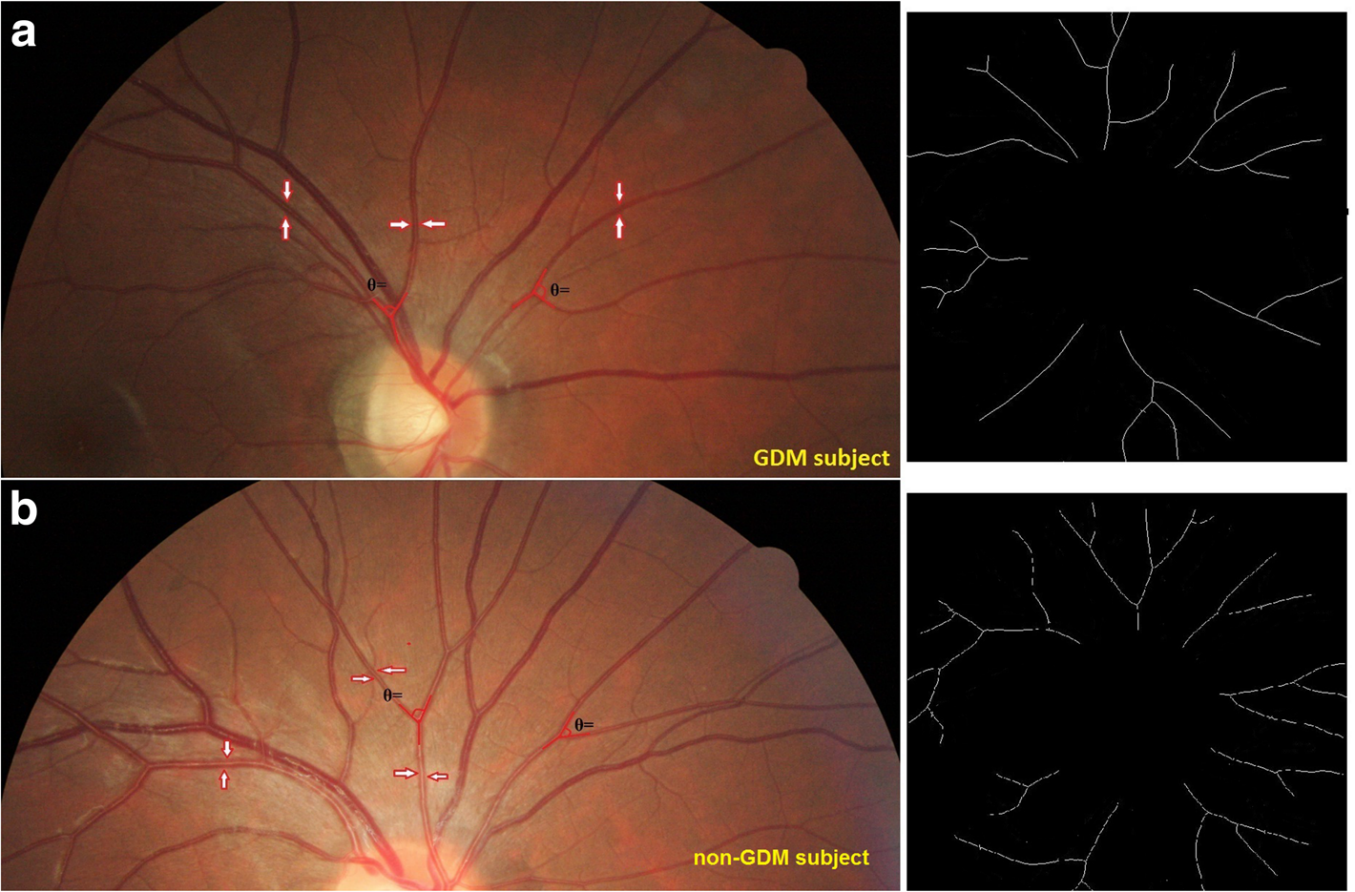
Comparison of retinal vasculature between a GDM (**a**) and a non-GDM GUSTO mother (**b**). Red arrows indicate retinal arterioles. Angle highlighted in red lines indicate retinal arteriolar branching angle. Black-and-white images on the right indicate retinal arteriolar fractal dimension. This GDM mother has narrower retinal arteriolar caliber (116.6 vs. 125.4 µm), narrower retinal arteriolar branching angle (1.26 vs. 1.31 Df), and lower retinal arteriolar fractal dimension (98.0 vs. 74.3 degrees) than the non-GDM mother

In multiple linear regression after adjusted for social demographic confounders including age, ethnicity, and maternal education (Table 3; Model 1), GDM subjects had narrower retinal arteriolar caliber (1.6 μm; 95% CI: –3.1 μm, –0.2 μm), reduced retinal arteriolar fractal dimension (–0.01 Df; 95% CI: –0.02 Df, –0.001Df), and larger retinal arteriolar branching angle (1.8°; 95% CI: 0.3°, 3.3°) than non-GDM subjects. After further adjustment for GDM risks in Model 3 (Table 2), the associations of GDM and retinal arteriolar caliber and fractal dimension were attenuated, while that of GDM and larger retinal arteriolar branching angle remained (2.0°; 95% CI: 0.5°, 3.6°) (Table 3, Model 2). An example with all these differences in retinal microvascular measures between GDM and non-GDM mothers were shown in Fig. 2.

**Table 3:** Associations between retinal microvascular parameters and gestational diabetes mellitus (GDM) in GUSTO cohort

Retinal Microvascular measures		Mean Difference between GDM and non-GDM Groups			
		Model 1		Model 2	
		Mean difference	95% CI	Mean difference	95% CI
Caliber (μm)	Arterioles	**-1.6**	**-3.1, -0.2**	-1.2	-2.7, 0.3
	Venules	-1.6	-3.6, 0.5	-1.3	-3.4, 0.8
Fractal dimension (Df)	Arterioles	**-0.01**	**-0.02, -0.001**	-0.01	-0.02, 0.001
	Venules	-0.01	-0.01, 0.001	-0.01	-0.01, 0.001
Branching angle (degrees)	Arterioles	**1.8**	**0.3, 3.3**	**2.0**	**0.5, 3.6**
	Venules	-0.8	-2.3, 0.8	-0.6	-2.2, 0.9

Model 1, adjusted for age, ethnicity and maternal education

Model 2, model 1 and additionally adjusted for history of hypertension, weight gain at 26–28 weeks gestation, family history of diabetes, maternal pre-pregnancy overweight/obese status, history of past gestational diabetes and parity

Bold data mean difference and 95% CI, meaning the *p* value for such estimates are significant

*Abbreviations: CI* confidence interval

No evidence was found for effect modification or interaction between exposure variables in our study.

## Discussion

In this study of Singaporean mothers, abnormal retinal arteriolar measures, including narrower arteriolar caliber, reduced fractal dimension, and larger branching angle, were observed in expectant mothers with GDM diagnosed at 26–28 weeks of gestation. Even after statistical adjustment for conventionally recognized risk factors of GDM, the association and statistical significance between GDM and retinal arteriolar branching angle persisted.

Small-vessel dysfunction has been shown to be a consequence of T2DM [8, 17], possibly as a result of endothelial dysfunction and inflammation resulting from chronic hyperglycemia [18, 19]. Hyperglycemia leads to vasoconstriction, reduces blood flow and exacerbates tissue hypoxia, with eventual compensatory vasodilation [7, 20]. Owing to the non-invasive and reproducible nature of the retinal microvasculature imaging, changes in retinal vascular geometry have been extensively studied and validated as proxies for small-vessel dysfunction, particularly in diabetes and other microvascular disorders [8].

Pregnancy is characterized by an altered inflammatory profile compared to the non-pregnant state, with a fine balance between pro- and anti-inflammatory cytokines needed for normal fetal development [21]. In up-regulated inflammatory conditions like obesity, this balance is altered and may compromise normal pregnancy [22]. Even though the pathophysiology of GDM is not fully understood, GDM is associated with the up-regulation of leptin and pro-inflammatory cytokines (e.g., interleukin-6 and tumor necrosis factor α) [23, 24]. Therefore, a heightened inflammatory response imposed by GDM during pregnancy may affect adipose tissue, placental and vascular endothelium, which in turn might lead to adverse clinical outcomes such as pre-eclampsia [25].

We found that abnormalities in retinal arterioles, but not in retinal venules, were associated with GDM. These microvascular changes include narrower retinal arteriolar caliber, reduced retinal arteriolar fractal dimension, and larger retinal arteriolar branching angle. Since hyperglycemia is presumed to have an initial vaso-constrictive effect, followed by irreversible vasodilatation [7], the same principle may apply in GDM: transient hyperglycemia results first in vasoconstriction and is reflected by retinal arteriolar narrowing.

We also assessed retinal arteriolar geometry in terms of branching angle and fractal dimensions. The larger the branching angle, the greater the workload and energy spent in maintaining efficient blood circulation [26]. Larger branching angles have been linked to atherosclerosis [27], disrupted blood flow [28], and endothelial damage [29]. Fractal dimension, on the other hand, quantifies the degree of geometric complexity, with reduced fractal dimension reflecting a sparser branching pattern and lower vascular structural complexity [30]. We found that both reduced retinal arteriolar fractal dimension and larger retinal arteriolar branching angle were associated with GDM. These morphological abnormalities may have been due to the hypoxic state of the retina consequent to a hyperglycemic environment in GDM.

Similar to retinal imaging studies obtained in patients with diabetes, we postulate that investigating microvascular changes in GDM may provide valuable insights into such pregnancy complications. To the best of our knowledge, ours is the first study demonstrating that changes in the maternal retinal microvasculature, specifically the retinal arterioles, are associated with GDM and may reflect the role of small-vessel dysfunction as its pathophysiological consequence.

Strengths of our study include a large sample size of pregnant women and GDM cases, standardized protocols using validated assessments of GDM and retinal vasculature, and detailed information on a range of potential confounders and risk factors for GDM. However, several methodological limitations are also noted. First, only approximately half of all recruited subjects were included in our analyses, and selection bias cannot be excluded. However, as shown in Table 1, mothers with and without retinal vessel assessments were similar in baseline factors, and it seems likely that our results are generalizable to our overall study cohort. Second, our study was cross-sectional, hence the causal relationship between GDM and retinal microvascular changes warrants further investigation. In some studies, retinal arteriolar narrowing and impaired microvascular perfusion have been suggested to delay the access of glucose and insulin to target tissues, which leads to insulin resistance—a major mechanism underlying type 2 diabetes [31]. Third, measurement error may have occurred in maternal recall of covariates such as pre-pregnancy weight, past history of GDM, and family history of diabetes. Any such errors, however, are likely to have been non-differential with respect to the microvascular changes we studied, and thus would tend to bias true associations toward the null.

## Conclusions

In summary, we observed abnormalities in the retinal arteriolar microvasculature, including narrower caliber, reduced fractal dimensions and larger branching angle, in expectant mothers with GDM during the late second trimester of pregnancy. Further research is required to investigate whether the vascular changes we observed persist after pregnancy, and whether they predict the later development of type 2 diabetes or cardiovascular disease.

## Abbreviations

BMI: Body mass index
CI: Confidence interval
GDM: Gestational diabetes mellitus
GUSTO: Growing Up in Singapore Towards healthy Outcomes
KKH: KK Women’s and Children’s Hospital
NUH: National University Hospital
OGTT: Oral glucose tolerance test
SIVA: Singapore I vessel analyzer
T2DM: Type 2 diabetes mellitus
WHO: World Health Organization
